# HILIC-MS for Untargeted Profiling of the Free Glycation Product Diversity

**DOI:** 10.3390/metabo12121179

**Published:** 2022-11-25

**Authors:** Yingfei Yan, Daniel Hemmler, Philippe Schmitt-Kopplin

**Affiliations:** 1 Research Unit Analytical BioGeoChemistry (BGC), Helmholtz Zentrum München, Ingolstädter Landstrasse 1, 85764 Neuherberg, Germany; 2 Comprehensive Foodomics Platform, Chair of Analytical Food Chemistry, TUM School of Life Sciences, Technical University Munich, Maximus-von-Imhof-Forum 2, 85354 Freising, Germany

**Keywords:** non-enzymatic glycation, Maillard reaction products, HILIC-MS, untargeted analysis, advanced glycation end products

## Abstract

Glycation products produced by the non-enzymatic reaction between reducing carbohydrates and amino compounds have received increasing attention in both food- and health-related research. Although liquid chromatography mass spectrometry (LC-MS) methods for analyzing glycation products already exist, only a few common advanced glycation end products (AGEs) are usually covered by quantitative methods. Untargeted methods for comprehensively analyzing glycation products are still lacking. The aim of this study was to establish a method for simultaneously characterizing a wide range of free glycation products using the untargeted metabolomics approach. In this study, Maillard model systems consisting of a multitude of heterogeneous free glycation products were chosen for systematic method optimization, rather than using a limited number of standard compounds. Three types of hydrophilic interaction liquid chromatography (HILIC) columns (zwitterionic, bare silica, and amide) were tested due to their good retention for polar compounds. The zwitterionic columns showed better performance than the other two types of columns in terms of the detected feature numbers and detected free glycation products. Two zwitterionic columns were selected for further mobile phase optimization. For both columns, the neutral mobile phase provided better peak separation, whereas the acidic condition provided a higher quality of chromatographic peak shapes. The ZIC-cHILIC column operating under acidic conditions offered the best potential to discover glycation products in terms of providing good peak shapes and maintaining comparable compound coverage. Finally, the optimized HILIC-MS method can detect 70% of free glycation product features despite interference from the complex endogenous metabolites from biological matrices, which showed great application potential for glycation research and can help discover new biologically important glycation products.

## 1. Introduction

Non-enzymatic glycation has received increasing attention over the past few decades in both food chemistry and in vivo studies [1]. This type of reaction was initially discovered by a French chemist, Louis C. Maillard, in 1912, referring to the reaction between reducing carbohydrates and amino compounds. The spontaneous condensation reaction between the amino group and carbonyls first forms unstable Schiff bases, which rearrange to more stable Amadori products (ARPs). Consecutive degradation of ARPs produces highly reactive dicarbonyls, such as deoxyosone, glyoxal, methylglyoxal, etc. Dicarbonyls react with the amino group and yield advanced glycation end products (AGEs). For instance, the reactions between glyoxal, and methylglyoxal with lysine form carboxymethyl-lysine (CML), and carboxyethyl-lysine (CEL), respectively, which are often used as makers for the Maillard reaction (MR). Eventually, a heterogeneous mixture of Maillard reaction products (MRPs) with diverse structures is produced from minor initial precursors through complex consecutive reaction cascades [2,3].

MR not only contributes to the aroma, taste, and color for foods during thermal processing, but also has close biological associations with aging and diseases, such as chronic hyperglycemia, diabetes, etc. The accumulation of endogenous free glycation products usually indicates metabolic disorders in vivo. Increased levels of free AGEs in plasma were associated with increased levels of diabetes complications [4,5]. Phenylalanine-glucose ARP was found as a biomarker for phenylketonuria, an inherited disorder causing the build-up of phenylalanine in the body [6]. The accumulation of free glycerate-modified amino acids, forming through the non-enzymatic reaction between amino acids and a highly reactive glycolytic intermediate, was detected in the brain of Parkinson’s disease protein PARK7 knockout mouse [7]. The above-mentioned points suggested the important biological roles of endogenous AGEs and that a reliable method for analyzing these free glycation products is a basis to reveal their functions.

Common methods for glycation product analysis involve enzyme-linked immunosorbent assay (ELISA) and analytical instruments, including LC-MS [8,9,10] and GC-MS [11]. Among these methods, LC-MS provides a sensitive, selective, and high-throughput analysis without the need for specific antibodies or derivatization. Based on the structural prototypes, relevant in vivo AGEs can be classified into various categories depending on the amino acids involved and if crosslinks between amino acid residues occur [12]. AGEs with lysine residues, arginine residues, and crosslinks between two residues are the most investigated, such as CML, CEL, glyoxal-hydroimidazolone (G-H), methylglyoxal-hydroimidazolone (MG-H), glyoxal-lysine dimer (GOLD), and pentosidine. Up to twenty selected free glycation products, including ARPs and AGEs, could be quantified in targeted approaches in biological matrices, such as plasma, saliva, and urine [13,14]. However, AGEs have very diverse structures and biologically important glycation products are not always these well-studied AGEs. Thus, there is a need to establish a reliable method for comprehensively qualifying and quantifying free glycation products in biological samples, including the many hitherto unknown AGEs. Commercially available glycation product standards are very limited in number and structural diversity. Moreover, the synthesis of AGE standards is very time-consuming and challenging. Maillard model systems are valuable alternatives to limited standards for method development. They can reproducibly produce a mixture of hundreds of different free glycation products from only a few initial precursors with low-cost [15,16]. Although some reaction pathways for forming these glycation products are different in vivo compared with model systems [1], most glycation compounds can also be formed in model systems. Therefore, in current study, we used the MR model system to optimize the method based on untargeted strategy rather than using a limited number of commercially available reference standards.

Most free glycation products are highly polar and, therefore, have limited retention on reverse phase (RP) columns which are the most commonly used stationary phase in LC. To solve this issue, ion-pair reagents, such as heptafluorobutyric acid, nonafluoropentanoic acid were often used in previous studies to provide enough retention and efficient separation for free glycation products on RP columns [8,9,17]. However, ion-pair reagent has several drawbacks, including ionization suppression for mass spectrometry (MS), contamination of the instrument, and reduced column lifetime. Hydrophilic interaction liquid chromatography (HILIC) is a promising option for analyzing polar compounds. Until now, very limited studies used HILIC for the quantification of AGEs and they all focused on the targeted quantification of specific AGEs in foods [18,19,20]. To the best of our knowledge, systematic evaluation of HILIC columns and conditions for profiling free glycation products in an untargeted way is still lacking.

This study aimed to establish a method for the simultaneous characterization of diverse free glycation products using an untargeted metabolomics approach. Five different HILIC columns were compared. The mobile phase composition was further investigated to maximize performance and robustness of the analytical method. Methods were evaluated in terms of the number of detected features, distribution of detected features, precision, number of detected known glycation products, and their peak shapes. The ability of the optimized method was finally evaluated using biological matrices such as plasma, feces, and urine.

## 2. Materials and Methods

### 2.1. Reagents and Materials

Twenty proteinogenic L-amino acids (>97%), L-cystine (≥98%), D-(+)-glucose (≥99.5%), acetic acid (LC-MS grade), ammonium formate (10 M stock solution), and ammonium acetate (5 M stock solution) were purchased from Sigma–Aldrich (Steinheim, North Rhine-Westphalia, Germany). Acetonitrile (ACN, LC-MS grade) was purchased from Merck (Darmstadt, Hesse, Germany). Formic acid (98%, for mass spectrometry) was ordered from Honeywell Fluka (Charlotte, NC, USA). Purified water (18.2 MΩ) was obtained from a Milli-Q integral water purification system (Billerica, MA, USA). ESI-L low-concentration tuning mix was supplied by Agilent (Santa Clara, CA, USA). Lyophilized human plasma (P9523) was purchased from Sigma-Aldrich (Saint Louis, MO, USA) and reconstituted with double distilled water. One feces and urine sample was collected from a healthy male volunteer. Plasma, feces, and urine samples were kept frozen at −80 °C until use.

### 2.2. Maillard Model Systems Preparation

Twenty-one amino acids were reacted with glucose, respectively, to obtain MRPs with a wide range of physicochemical properties. Equal molar mixtures of glucose (0.1 M) and each amino acid (0.1 M) were prepared in Milli-Q water and heated in closed glass vials at 100 °C for seven hours to make Maillard model system samples. The same volume of each model system was mixed together, referred to as the model systems mixture sample (MSM1), and then diluted by 1:5 (*v*/*v*) with 90% acetonitrile for LC-MS/MS analysis. Model system samples of lysine (Glc-Lys), arginine (Glc-Arg), and histidine (Glc-His) were diluted 1:10 (*v*/*v*) with 90% acetonitrile for analysis. All samples were analyzed in triplicate.

### 2.3. Biological Sample Preparation

Biological sample extracts were mixed with model systems to evaluate the optimized HILIC-MS method. Plasma, urine, and feces samples were thawed on ice and vortexed for 60 s before sample preparation. 25 μL plasma was mixed with 500 μL ACN and vortexed for 5 min. 50 μL urine was diluted using 1 mL ACN and stored on ice for 5 min. The feces sample was first centrifuged at 14,000× *g* rpm at 4 °C for 10 min. Thereafter, approximately 50 mg of the pellet was homogenized with ACN at a fixed ratio of 1:20 (mg/mL) and sonicated in an ice bath for 30 min. All samples were then centrifuged at 14,000× *g* rpm at 4 °C for 10 min. The supernatants were transferred to another tube and then mixed with the model system solution later.

Equal volumes of Glc-Lys and Glc-Arg were mixed to get a model system mixture (named as MSM2). Before being spiked with biological extracts, MSM2 was mixed with water to obtain the low (1:25, *v*/*v*), medium (1:5, *v*/*v*), and high (undiluted) model system solution. In total, 90 μL of each type of biological extract supernatant was mixed with 10 μL of MSM2 solution in three concentration levels separately for matrix effect evaluation. The biological extracts without MSM2 (90 μL biological extracts mixed with 10 μL water) and MSM2 without biological extracts (10 μL MSM2 mixed with 90 μL ACN) were used as controls.

### 2.4. LC-MS/MS

Samples were analyzed using a Waters Acquity (Milford, MA, USA) UPLC system coupled with a Bruker maXis Quadrupole time-of-flight (QTOF) MS (Bremen, Germany). The injection volume was 5 μL for each sample. The MS analyses were performed in positive electrospray mode with a mass range of 50–1500 *m/z*. The ion source settings were: nebulizer gas pressure 2 bar, capillary voltage 4500 V, dry gas flow 10 L/min, and dry gas temperature 200 °C. Mass spectra were acquired with a scan rate of 5 Hz in data-dependent mode, where the three highest MS1 ions of each precursor scan were chosen for MS/MS with a collision energy of 30 eV. The TOF analyzer was calibrated using ESI-L Low Concentration Tuning Mix (Agilent, Santa Clara, CA, USA). Additionally, the same diluted Tuning Mix (1:4 (*v*/*v*) with 75% ACN) was injected from 0.1 to 0.3 min of every measurement using a switching valve for internal recalibration.

### 2.5. Chromatographic Conditions Optimization

This study aimed to establish a method for analyzing free glycation products instead of in-depth investigating the mechanisms of HILIC. Therefore, to find appropriate parameters in a straightforward and time-consuming manner, we chose a univariate method optimization approach, rather than testing all factors at all levels.

Firstly, five different HILIC columns were tested: iHILIC-Fusion (100 × 2.1 mm, 1.8 μm, 100 Å, zwitterionic, HILICON AB, Umea, Sweden), ZIC-cHILIC (100 × 2.1 mm, 3 µm, 100 Å, zwitterionic, Merck, Darmstadt, Germany), ZIC-HILIC (100 × 2.1 mm, 3.5 µm, 100 Å, zwitterionic, Merck, Darmstadt, Germany), BEH-HILIC (150 × 2.1 mm, 1.7 µm, 130 Å, unbonded ethylene bridged hybrid (BEH) particle substrates, Waters, Eschborn, Germany), BEH-Amide (100 × 2.1 mm, 1.7 µm, 130 Å, BEH amide, Waters, Eschborn, Germany). All columns were compared using the same eluents and gradient based on the recommendation of our previous study [21]. Eluent A consisted of 25 mM ammonium acetate (AA) in 30% ACN (pH 4.6), and Eluent B consisted of 5 mM AA in 95% ACN (pH 4.6). The binary gradient was: 0 min, 99.9% B; 2 min, 99.9% B; 9.5 min, 0.1% B; 12 min, 0.1% B; 12.1 min, 99.9% B. Columns were equilibrated at 99.9% B for 3 min after each run to reach the initial status. The flow rate was 0.5 mL/min with the column temperature set at 40 °C.

After the selection of the optimum stationary phase for analyzing MRPs, the effect of pH and mobile phase additives were further evaluated to choose the optimum mobile phase composition. To avoid ion suppression caused by high salt concentration, 5 mM ammonium formate (AF) or AA was used as the mobile phase modifier. Mobile phases A consisted of 5 mM salt in 30% ACN (acidic: with 0.1% corresponding acid; neutral: without corresponding acid) and mobile phases B consisted of 5 mM salt in 95% ACN (acidic: with 0.1% corresponding acid; neutral: without the corresponding acid) was tested. The detailed information for mobile phase optimization is shown in Table S1. Finally, the gradient was improved using the optimal mobile phase. The ultimate chromatographic condition was: eluent A 5% ACN and eluent B 95% ACN, both with 5 mM AF and 0.1% formic acid (FAcid). The gradient was: 0 min, 99.9% B; 2 min, 99.9% B; 13 min, 56% B; 14 min, 30% B; 14.1 min, 10% B; 16 min, 10% B; 16.1 min, 99.9% B.

### 2.6. Data Processing

Raw data were calibrated and converted to mzXML files by Bruker DataAnalysis 5.0 software (Bremen, Germany). The data preprocessing of the converted files, including peak picking, peak alignment, peak correspondence, and MS2 spectra finding were done based on the XCMS package (4.1.2) in R (version 4.1.0) [22]. The feature grouping, isotopes finding, and adducts annotation were processed by the CAMERA package (version 4.1.1) [23]. The detailed settings for XCMS and CAMERA are shown in Tables S2 and S3, respectively. The in-source fragment (ISF)-finding algorithm was adapted from the ISFrag package (version 0.1.0) [24]. Feature cleaning was carried out by in-house script in R. Principal component analysis (PCA) was completed using the FactoMineR package (version 2.4) [25].

## 3. Results

In this study, several chromatographic conditions for the analysis of free glycation products were evaluated through untargeted and targeted comparisons using typical MR model systems. The capability of the optimized method was further assessed by analyzing model systems spiked with biological matrices, including plasma, urine, and feces.

### 3.1. Data Cleaning for Reliable Feature Lists

The main aim of this study is to establish a method for simultaneously characterizing a wide range of free glycation products using the untargeted metabolomics approach. In contrast to the common method development workflow, where the occurrence of fixed reference standard compounds was compared, complex MR systems were chosen for method optimization. As reported by previous studies, the model system consisting of single amino acids and sugar can produce a multitude of glycation products [15,16], which has better coverage of potential glycation products compared to using the very limited commercially available glycation standards. Using the untargeted approach to analyze the model system is capable of generating an overall description of compounds in the MR mixture. Besides MRPs, there are also amino acid, glucose, and minor degradation products of reactants in the model system. The feature list created from the untargeted data processing algorithm contains redundant peaks derived from one compound, including isotope peaks, ISFs, various adducts, multimers, containments and artifacts, which makes the number of features directly derived from peak detection not accurate for method comparison.

**Figure 1:**
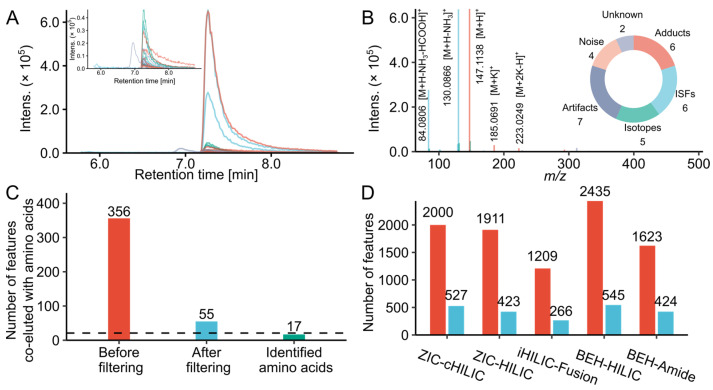
Feature filtering results of model systems. (**A**) Extracted ion chromatograms of lysine and its co-eluted MS features grouped by CAMERA in a glucose and lysine model system analyzed by iHILIC-Fusion. (**B**) Interpretation of the MS features grouped with lysine. Top right: Pie chart of the proportions of peaks categorized by the annotations. (**C**) The number of features co-eluted and grouped with amino acids in the model systems mixture of twenty-one amino acids (MSM1) before and after filtering analyzed by iHILIC-Fusion. (**D**) The total feature number detected in MSM1 before (red) and after filtering (blue) analyzed by different HILIC columns.

Hence, we further attributed feature relationships by an in-house R script following the CAMERA annotation [23]. A typical output of MS feature annotations associated with one compound was exemplified with lysine. As shown in Figure 1A,B, there were 30 co-eluted features with the same chromatographic peak shape (Pearson correlation coefficient > 0.8, *p* < 0.001) in the correlation group containing the lysine [M + H]^+^ signal. Detailed information and interpretations of each feature are shown in Table S4. By using the current data processing workflow, the 30 features can be divided into 6 categories, including 4 low intensity noise, 5 isotope peaks, 7 artifacts caused by the saturation of the detector [26], 6 adducts, 6 ISFs, and 2 unidentified features. Among them, 22 features were produced by lysine. The approximately 95% feature inflation during untargeted LC-MS analysis was also reported in previous studies, 869 features were detected after the injection of 51 standards [27], and 10,000–30,000 features were observed by analyzing 900 unique metabolites [28].

To remove such unreliable and redundant features, we filtered feature lists by following steps: remove background ions, noise with low intensity, artifacts caused by saturation, isotope peaks, ISFs, redundant adducts. To evaluate the approach, we checked the features correlated with 21 amino acids in the MSM1 before and after filtering. As shown in Figure 1C, there were 356 features co-eluted and grouped with amino acids with high peak shape similarity before data cleaning. The filtering caused a reduction of 84.5% features, only 55 features were kept. Among these, 17 features were confirmed as amino acids. L-cysteine and L-aspartic acid were not detected because their intensities were below the limit of detection. L-leucine and L-isoleucine were not separated and identified as one compound. Only L-proline was not identified because it was incorrectly annotated as a potassium adduct. This indicates the current workflow can remove redundant features originated from same compounds and keep the real signal at the same time. The effect of the filtering process on whole datasets measured by different LC conditions was also compared and demonstrated using the MSM1 sample (Figure 1D). Around 22% to 26% of the total features were kept. The percentages of the overall removed feature detected in MSM1 were lower compared to the features co-eluted with amino acids. Because amino acids in the MSM1 produced more artifacts (~77% features caused by detector saturation related to amino acids) and redundant features (e.g., ISFs, multiple adducts) due to their high concentration. The feature inflation is less pronounced for most MRPs with relatively low concentration. Moreover, the data filtering did not cause significant alterations to the overall trends, suggesting the comparison results from feature number were acceptable.

### 3.2. Selection of HILIC Columns

#### 3.2.1. Non-Targeted Evaluation of the Column Selection

Three types of HILIC columns, including one bare silica (BEH HILIC), one amide (BEH Amide), and three zwitterionic columns (iHILIC-Fusion, ZIC-cHILIC and ZIC-HILIC), were selected for testing the selectivity of different stationary phases. According to our previous study on the thorough evaluation of metabolites coverage under different mobile phases, amino acids and their analogs preferred acidic conditions for both zwitterionic, and amide HILIC columns [21]. So, we used 30% ACN with 25 mM AA and 95% ACN with 5 mM AA at pH 4.6 as the starting mobile phase A and B to screen the columns.

We compared the performance of different columns by analyzing model systems. The aim was to maximize the number of detected MRP features, which were characterized by unique *m/z* and retention time with good reproducibility. The MSM1 was chosen for evaluating the selectivity and coverage of columns for MRPs that derivate from all proteinogenic amino acids, like the ARPs. In addition, lysine, arginine and histidine glucose model systems were analyzed separately. Because of the higher reactivity for these amino acids and N-containing side chains, most free AGEs reported in in vivo studies are derived from them [14,29].

**Figure 2:**
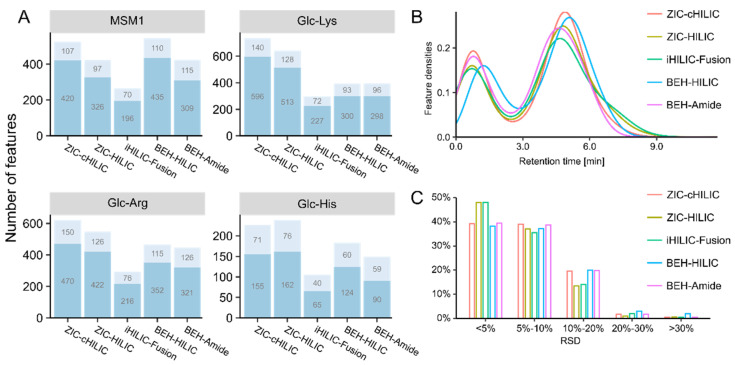
Results of column selection. (**A**) Bar plots representing the number of features detected by different columns in each model system. Features were categorized into two types: features with MS2 (light blue) and features without MS2 but recognizable extracted ion chromatograms (dark blue). MSM1: the model systems mixture of twenty-one amino acids. (**B**) Retention time distribution of all detected features in model systems. (**C**) Relative standard derivation (RSD) distribution of all feature intensities.

Results for features in each model system detected by the tested columns are summarized in Figure 2. The features were classified into two types: features with and features without MS2 spectra. Features with MS2 spectra promise both downstream statistical analysis and the possibility of compound structural identification. The higher number of MS2 spectra also suggests a better separation of LC when total feature numbers are comparable. Among all tested columns, ZIC-cHILIC detected the highest overall number of features using the same mobile phase (Figure 2A). Particularly for Glc-Lys, the feature number detected by ZIC-cHILIC was more than 1.5-fold higher than for other columns (except for ZIC-HILIC). Independent of the tested columns, more features were detected in Glc-Lys and Glc-Arg compared with the Glc-His model system, suggesting the higher reactivity towards Glc for Lys and Arg. However, features observed in MSM1 were lower than expected, and less than those in Glc-Lys and Glc-Arg. This could be ascribed to the low concentration of most MRPs in each model system and the high concentration of reactant amino acids.

The retention time (RT) density plot (Figure 2B) shows the distribution of all features detected in four model systems by different HILIC columns along the RT. For accurate quantitation and simpler spectra complexity, it is preferable for fewer features to elute during the void volume. All HILIC columns showed good retention for detected features, resulting in a higher density between 3 min and 9.5 min compared with a RT of less than 2.5 min. Features were eluted ~0.35 min later for BEH-HILIC compared with other columns because BEH-HILIC has a greater column length (150 mm) and, consequently, a larger void volume. In general, ZIC-HILIC and iHILIC-Fusion columns showed more dispersed separation for features across the RT and fewer features eluting between 0 and 2 min. The precision was evaluated by calculating the relative standard derivation (RSD) of the intensity of the three analytical replicates for all detected features. As shown in Figure 2C, the RSD for more than 95% of features was less than 20%, showing good reproducibility for all tested columns.

#### 3.2.2. Selectivity of Columns for Analyzing Amino Acids and Glycation Products

**Figure 3:**
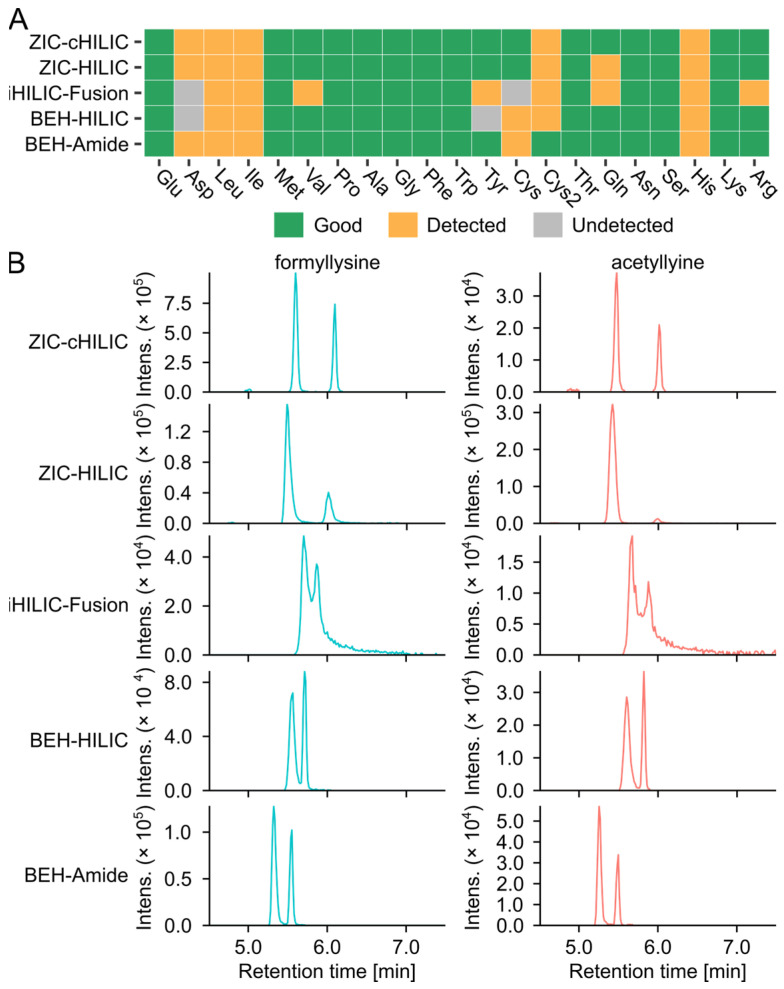
Targeted evaluation of column performance. (**A**) The individual score of amino acids analyzed by different HILIC columns. (**B**) Representative chromatograms of the putative advanced glycation end products isomers, formyllysine (C_7_H_14_N_2_O_3_, [M + H]^+^ = 175.1077, blue) and acetyllysine (C_8_H_16_N_2_O_3_, [M + H]^+^ = 189.1234, red), separated by different columns. EICs were extracted with ±0.005 Da.

Amino acids and known glycation products were then subjected to a detailed comparison (Figure 3). The ability of HILIC columns for analyzing amino acids in MSM1 was evaluated based on the retention time, peak shape, and MS intensity. All amino acids can be detected with good retention using ZIC-HILIC, ZIC-cHILIC, and BEH-Amide. Cystine, which is the oxidized dimer of cysteine, can only be detected with good peak shape and sensitivity by BEH-Amide. The peak width of amino acids analyzed by ZIC-HILIC and iHILIC-Fusion was broader compared with the other three columns and tended to tail especially for basic amino acids (Table S5). This is likely due to the stronger electrostatic attraction between the net positive charge of basic amino acids and negatively charge at the distal end of sulfobetaine in ZIC-HILIC [30], and the slightly negative net surface charge in iHILIC-Fusion [31], respectively.

The free glycation products, including AGEs and ARPs, that can be produced by the model systems were collected from the literature to build a library (Table S6). The analytical capability of the five tested HILIC columns was also benchmarked by the detectability of these glycation products. We screened the feature table of each column for glycation products using the theoretical *m*/*z* with an error < 10 ppm. Considering that the MR can produce multiple isomers of glycated amino acids, including stereoisomers, regioisomers, and anomers [32], all the isomers were summed to compare the number of matching features per unique *m*/*z* (Table S7). The ARPs were barely detected in the MSM1 and were not evaluated in the column selection section. This could be due to the ionic suppression caused by the high concentration of salts in the aqueous mobile phase (25 mM AA) [31,33]. Three types of zwitterionic columns can detect higher glycation candidates than bare HILIC and amide columns. ZIC-HILIC detected the highest number of potential AGEs features (24 matched features of 15 unique *m*/*z*) in Glc-Lys and GLc-Arg, followed by ZIC-cHILIC, 23 matched glycation product candidates and 12 unique *m*/*z*. In comparison, only 12 matched candidates (9 unique *m*/*z*) were detected with BEH HILIC. Glyoxal-lysine dimer (GOLD) and methylglyoxal-lysine dimer (MOLD) can be only detected by ZIC-HILIC. Representative extracted ion chromatograms (EICs) of AGEs are shown in (Figure 3B) to present the selectivity and performance of the column. The best separation of isomers of formyllysine and acetyllysine was achieved by the ZIC-cHILIC column.

Altogether, ZIC-HILIC and ZIC-cHILIC showed better fits for analyzing free glycation products through the column selection. These two columns were compared in detail for later optimization with a lower salt concentration in mobile phases.

### 3.3. Mobile Phase Optimization

#### 3.3.1. Non-Targeted Evaluation of the Mobile Phase pH and Modifiers

**Figure 4:**
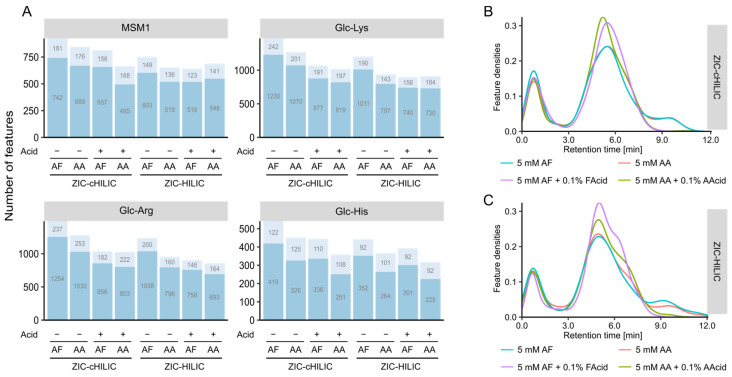
Effects of mobile phase composition on feature coverage and distribution. (**A**) Bar plots representing the number of detected features analyzed by mobile phases consisted of 5 mM ammonium formate (AF) or 5 mM ammonium acetate (AA) with and without its corresponding acid (0.1%, *v*/*v*). (**B**) Density distribution of retention times across the chromatographic run for ZIC-cHILIC column and (**C**) ZIC-HILIC column.

We next compared the performance of ZIC-cHILIC and ZIC-HILIC columns under neutral and acidic mobile phase conditions for two different modifiers (AF and AA), respectively. Among the eight investigated conditions, the ZIC-cHILIC column provided the highest feature number for all four samples using 5 mM AF as modifiers under the neutral condition (Figure 4A). Interestingly, ZIC-cHILIC always detected more features than ZIC-HILIC independent of the mobile phase condition and sample type. This could be due to the smaller particle size of the ZIC-cHILIC column (3 µm) compared with the ZIC-HILIC (3.5 µm) resulting in better separation performance [30]. Moreover, ZIC-cHILIC was reported to have better selectivity for polar amino-containing compounds than ZIC-HILIC, like aminoglycosides [34]. The feature count increased in all four model systems using neutral rather than acidic mobile phases. Based on the retention time distribution (Figure 4B,C), both ZIC-cHILIC and ZIC-HILIC showed improved separation under neutral conditions compared to acidic conditions. The better separation also explained the higher feature number at neutral mobile phases. Same results were also reported in previous literature [30,35,36], which may be attributed to the decreased ion-exchange interaction for both ZIC-HILIC and ZIC-cHILIC caused by the protonation of silanols with acidic mobile phases [37]. For the effect of additives, independent of the existence of the corresponding acid, a higher feature count was observed for AF compared with AA. The feature number detected in Glc-Lys and Glc-Arg model systems was higher than Glc-His, which was consistent with previous column selection results. Overall, the mobile phase composition had a similar impact on the feature number detected in each of the four tested model systems.

The precision of the tested conditions is shown in Figure S1. All conditions showed good precision with more than 95% features observed with an intensity RSD below 20%. The highest percentage of features with RSD less than 20% was observed for the ZIC-HILIC with 5 mM AF and 0.1% FAcid. For the features with less precision (RSD > 20%), a slightly higher percentage was observed under the neutral condition compared to the acidic condition.

#### 3.3.2. Effect of Mobile Phase on Detections of Amino Acids and Glycation Products

**Figure 5:**
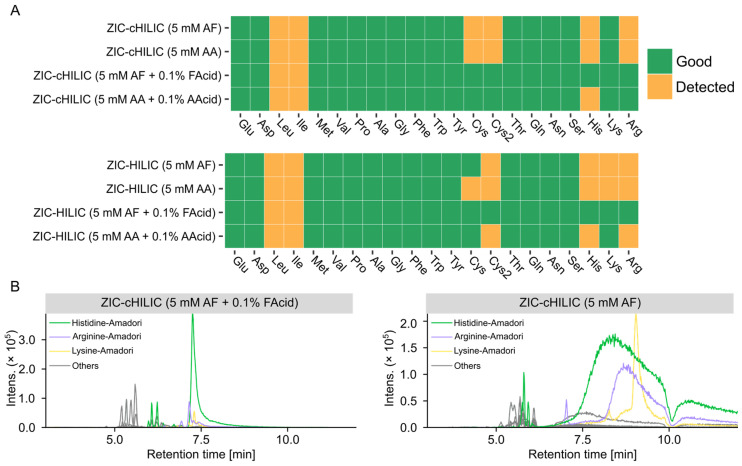
Effects of mobile phase on peak shapes of the amino acids and ARPs. (**A**) Individual score of amino acids analyzed by different mobile phases. (**B**) Extracted ion chromatograms of all 21 ARPs under acidic and neutral conditions (extracted with theoretical [M + H]^+^ ± 0.005 Da, detailed information of theoretical *m/z* is shown in Table S6).

The performance of all tested chromatographic conditions for analyzing amino acids is summarized in Figure 5A and Table S8. ZIC-cHILIC and ZIC-HILIC operated with mobile phase containing 5 mM AF and 0.1% FAcid can detect the highest number of amino acids with good peak shape. Mobile-phase pH has a higher impact on the peak shape of amino acids compared to column chemistry. Basic amino acids, including Lys, Arg, and His, have broad peaks under neutral conditions. One reason is the electrostatic attraction between the positively charged side chain of basic amino acid and the negatively charged silica under the neutral condition [31]. For His, the pKa of its side-chain group is 5.97. Under the neutral condition, the side chain of His is half deprotonated causing the broad peak shape. Cys and Cys2 were detected with good peak shape only under acidic conditions as well. The baseline separation of Ile and Leu was not achieved for all tested methods; however, a better separation was obtained by ZIC-cHILIC compared with ZIC-HILIC, which merged into one peak analyzed by ZIC-HILIC under most tested mobile phases.

ZIC-cHILIC operated under mobile phase containing 5 mM AF showed the best coverage for glycation products because 71 glycation product candidates could be matched (Table S9). Generally, more matched glycation candidates were observed under neutral conditions compared to acidic conditions, showing similar trends as revealed by the above performed comparison of untargeted feature numbers. This could be explained by the better separation of the column operated under neutral conditions and more isomers could be resolved. However, when we checked the peak shape of detected glycation products, we found most ARPs showed broad and split peaks under the neutral mobile phase (Figure 5B), which may be due to the equilibrium between Amadori product anomers [38]. Particularly for ARPs of basic amino acids, the EICs were extremely board analyzed by neutral mobile phase. Poor peak shapes tend to interfere with the peak picking algorithm and lead to unreliable peak detection results, but also more easily cause column carryover and interfere with later analysis.

Considering both quantity and quality of detected features, the ZIC-cHILIC operated under 5 mM AF and 0.1% FAcid provided the best results for amino acid glycation product discovery in terms of peak shape and compound coverage. Finally, the gradient was optimized based on the feature distribution along the chromatographic run. As most of the compounds eluted from 4 min to 7.5 min (Figure 4B), in order to achieve a better separation, a longer gradient was used to change mobile phase composition from 25% B to 75% B. The final gradient program is listed in Section 2.

### 3.4. Evaluation of the Optimized HILIC-MS Method Using Biological Samples

Compared with model systems, biological samples contain complex endogenous metabolites, large amounts of salts (urine), high diversity of lipids (plasma), and gut microbiota metabolites (feces). These metabolites and salts can affect both chromatographic separation and electrospray ionization [39,40]. Therefore, we further evaluated the performance of the optimized method on analyzing glycation products with complex biological extracts. Free endogenous glycations are aggravated by hyperglycemia, oxidative stress, and other metabolic diseases, whereas glycation products in the biological samples from healthy individuals are subtle without considering the dietary intake of AGEs [1,9,14]. Thus, we used the mixtures of model systems and biological extracts to investigate the effect of biological matrices on HILIC-MS analysis as proof of concept. To avoid detector saturation caused by reactants (amino acids and glucose) and keep the glycation products with low intensity detectable, only the mixture of lysine and arginine model systems (MSM2) was used as an additive to plasma, urine, and feces at three concentration levels: low (25-fold diluted), medium (5-fold diluted) and high (not diluted). For method evaluation, three types of samples were analyzed, including biological sample extractions, MSM2, and MSM2-spiked biological sample extractions. The interference of endogenous compounds on the detectability of glycation products was evaluated by comparing the number of detected MRP features in MSM2 with and without biological extracts. The reproducibility of the method was visualized by PCA score plots.

**Figure 6:**
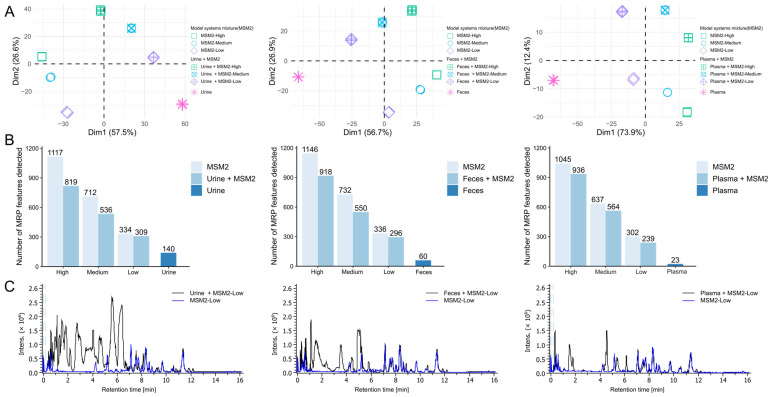
Detectability of glycation products in the biological matrices. (**A**) Principal component analysis (PCA) score plots of biological samples, a model system mixture (lysine and arginine; MSM2), and model system-spiked biological samples, in an order of urine, feces, and plasma. Prior to spiking, the model system mixture was either not diluted (**high**) or diluted 1:5 (**medium**) or 1:25 (**low**). Measurement was performed using the optimized HILIC method described in Section 2. (**B**) Bar plots representing the number of detected MRP features depending on the dilution level and biological extracts. (**C**) Representative base peak chromatograms of MSM2-Low and MSM2-spiked biological samples.

Based on PCA score plots (Figure 6A), three replicate injections of all sample types clustered together indicating good reproducibility. The first component discriminated the samples according to the concentration of spiked MRPs. For feces and plasma datasets, the samples spread from left to right along with the *x*-axis as their concentration increased, and the urine was the other way around. For all three PCA analyses, the first component can explain more than 50% of the variance, showing the optimized HILIC-MS method is capable of showing concentration differences of MRPs among samples. The second component discriminated the MSM2 samples versus those without biological extracts. The corresponding loading plots (Figure S2) indicate that most features were important in explaining the variability among different samples as they were positioned close to the correlation circle. In the loading plots, unique features detected in biological samples are located in the same region as the biological samples in the score plots (Figure 6A). Likewise, for MSM2 unique features in the loading plots were found in the same region as the MSM2 samples in the score plots (Figure 6A). The position of MSM2-spiked biological samples in score plots was in the middle position of biological samples-specific features and MSM2-specific features in loading plots. This supports the separation of groups of biological samples, MSM2 samples, and MSM2-spiked biological samples displayed in the score plots (Figure 6A).

Using human plasma, feces, and urine, we demonstrated that more than 70% of MRP features can be detected even in complex biological matrices (Figure 6B). Among these sample types, plasma has the least matrix effect compared with urine and feces: ~80% of MRP features can be detected for all three levels of mixture. The most suppression was caused by urine samples, only 73% MRPs could be recovered for the mixture with high concentration MSM2. This could be attributed to the more and higher concentration of polar compounds in urine and feces samples compared to plasma, resulting in ion suppression [41,42]. The chromatogram of plasma was relatively empty in contrast to feces and urine in our dataset (as shown in Figure 6C). Most MRPs eluted between 6 and 12 min. Representative EICs of AGEs and amino acids showed that the peak shape and isomer separation were not influenced by the in vivo metabolites (Figure S3). Interestingly, there are several features could be detected in both biological extracts and MSM2. We manually checked that some features were glucose, amino acids as well as their degradation products. Importantly, feces and urine had a higher number of matching features compared to plasma. This also explains the higher detection rate of MRP features in mixed samples at low levels compared to high levels. This suggested that urine and feces are better matrices for discovering glycation product candidates compared to plasma. Additionally, levels of free AGEs in urine and feces are more prone to be affected by dietary AGEs compared to plasma. More than 80% of dietary ARPs were not absorbed and degraded by gut microflora [43]. A significant increase of 40% free urinary CML excretion versus 7% higher plasma CML was detected in healthy people urine and plasma after the 2.5 times higher AGEs diet [44]. That should be considered during the experiment design and data interpretation of finding in vivo free glycation markers.

## 4. Conclusions

In the present study, we systematically optimized HILIC-MS methods for untargeted profiling of free glycation products using model systems. The performance of the methods was evaluated from both targeted and untargeted aspects. For untargeted comparison, the number of detected features, the distribution of features along the chromatographic window, and precision were assessed. The number of detected amino acids and matched known glycation products in model systems as well as their peak shapes were checked to further confirm the analytical ability of the method. With regard to the number of detected MRP features and matched AGEs, ZIC-HILIC and ZIC-cHILIC columns had better performance than the other three HILIC columns. Further mobile phase optimization of the selected two columns both showed neutral conditions can provide better peak separation and acidic conditions supported higher quality of chromatographic peak shapes. Considering the coverage and reproducibility, ZIC-cHILIC operated under an acidic condition with 5 mM AF and 0.1% FAcid was chosen as the final method. The performance of the optimized method with complex biological extracts proved that it still has good reproducibility and coverage for glycation products in the presence of endogenous metabolites from plasma, urine, and feces. Overall, the proposed method can be used to discover potential glycation markers associated with health and disease.

## Data Availability

The data presented in this study are available in the article and supplementary materials.

## Supplementary Materials

The following supporting information can be downloaded at: https://www.mdpi.com/article/10.3390/metabo12121179/s1, additional experimental details, data processing parameters, peak shape scores, and detected free glycation products (Tables S1–S9); the precision of feature intensities analyzed by different mobile phases, loading plots of principal component analysis, and representative EICs of amino acids and putative AGEs (Figures S1–S3).
