# Supplementary material for: HILIC-MS for Untargeted Profiling of the Free Glycation Product Diversity
Source: Metabolites. 2022 Nov 25;12(12):1179. doi: 10.3390/metabo12121179 (PMC9783660; doi:10.3390/metabo12121179)
Supplement: Supplementary file 1 [file metabolites-12-01179-s001.zip › metabolites-2042468-supplementary-proofread/metabolites-2042468-proofreading-supplementary.pdf]

**SUPPORTING INFORMATION**

# HILIC-MS for Untargeted Profiling of the Free Glycation Product Diversity

**Yingfei Yan [1,*], Daniel Hemmler [1,2] and Philippe Schmitt-Kopplin [1,2,*]**

[1] Research Unit Analytical BioGeoChemistry (BGC), Helmholtz Zentrum München, Ingolstädter Landstrasse 1, 85764 Neuherberg, Germany
[2] Comprehensive Foodomics Platform, Chair of Analytical Food Chemistry, TUM School of Life Sciences, Technical University Munich, Maximus-von-Imhof-Forum 2, 85354 Freising, Germany
* Correspondence: yingfei.yan@helmholtz-muenchen.de (Y.Y.); schmitt-kopplin@helmholtz-muenchen.de (P.S.-K.)

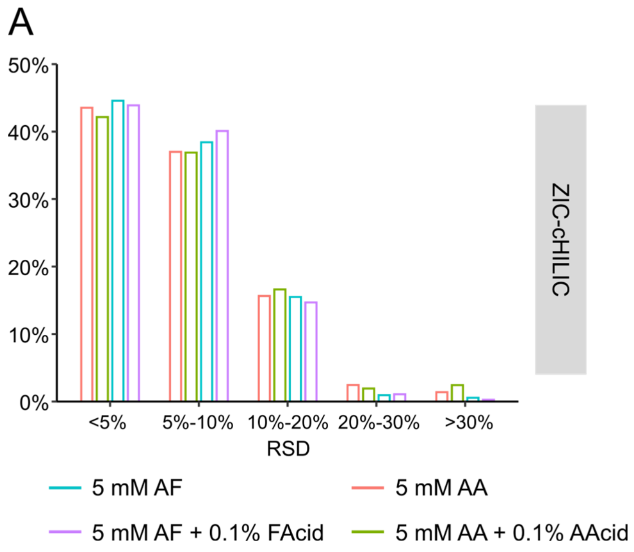


**Figure S1.** Relative standard derivation (RSD) distribution of all feature intensities detected by ZIC-cHILIC column (A) and ZIC-HILIC column (B) using different mobile phases.

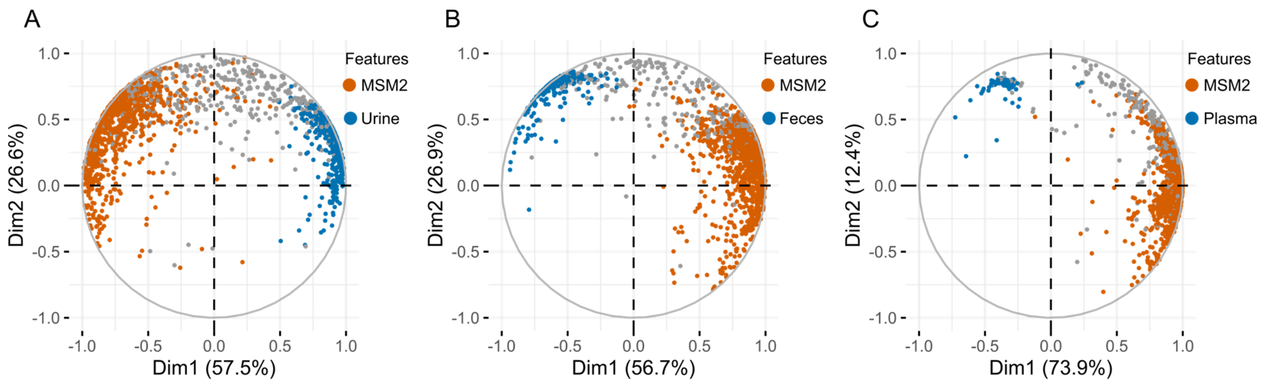


**Figure S2.** Principal component analysis (PCA) loading plots. Each loading plot corresponds to a PCA score plot in Figure 6A. In the loading plots, each point represents a feature with unique *m/z* and retention time. Points in blue refer to features that were only detected in biological samples: urine (A), feces (B) and plasma (C). Features in orange were only detected in model system mixture samples (lysine and arginine; MSM2).

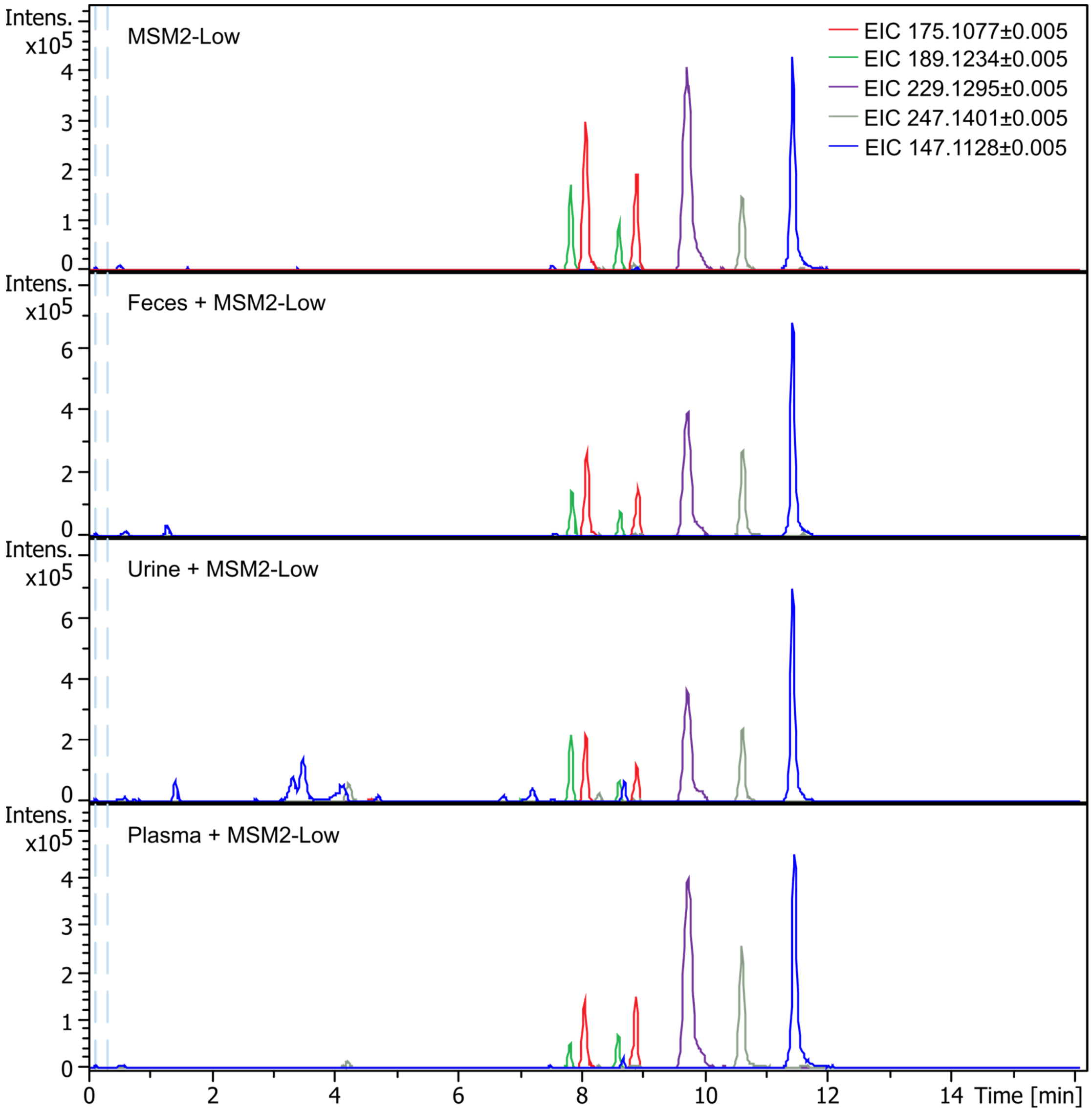


**Figure S3.** Representative extracted ion chromatograms (EICs) of amino acids and putative advanced glycation end products in the 25 times diluted model system mixture (lysine and arginine; MSM2), and model system-spiked biological samples; from top to bottom: model system mixture MSM2, MSM2 spiked feces, urine and plasma. EICs correspond to: formyllysine ($[M + H]^+$ = 175.1077, red), acetyllysine ($[M + H]^+$ = 189.1234, green), methylglyoxal hydroimidazolones ($[M + H]^+$ = 229.1295, purple), carboxyethylarginine ($[M + H]^+$ = 247.1401, grey), lysine ($[M + H]^+$ = 147.1128, blue).
